# Validation of the child and youth resilience measure among South African adolescents

**DOI:** 10.1371/journal.pone.0185815

**Published:** 2017-10-05

**Authors:** Kaymarlin Govender, Richard G. Cowden, Kwaku Oppong Asante, Gavin George, Candice Reardon

**Affiliations:** 1 Discipline of Psychology, School of Applied Human Sciences, University of KwaZulu-Natal, Durban, KwaZulu-Natal, South Africa; 2 Health Economics and HIV and AIDS Research Division, University of KwaZulu-Natal, Durban, KwaZulu-Natal, South Africa; 3 Institute of Psychology and Wellbeing, School of Psychosocial Behavioral Sciences, North-West University, Potchefstroom, North West, South Africa; TNO, NETHERLANDS

## Abstract

Resilience is a dynamic, interactive process between resources that contribute to safeguarding a person and the adversities they experience. Within this promotional framework of resilience, this study sought to validate the Child and Youth Resilience Measure (CYRM-28) among a sample of South African adolescents (*N* = 1854). Confirmatory factor analysis supported a superior level of fit for a 24-item, three-factor model (i.e., *individual/social*, *familial*, and *community/spiritual*). Internal consistency and test-retest reliability estimates at a 12-month interval (*N* = 648) supported the reliability of the scales. Higher scores on the scales were associated with feeling more connected at school, greater parental monitoring perceptions, and lower sexual risk, confirming the convergent and criterion validity of the instrument. Partial discriminative power was evidenced based on selected scale distinctions according to age and sex groupings. Collectively, the findings suggest the 24-item CYRM is a valid and reliable self-report measure to assess the availability of resources associated with resilience in South African youth.

## Introduction

Despite continuing debate on how best to define resilience and the variety of approaches that are used to study the construct, resilience is typically viewed as the capacity to successfully adapt to disturbances (e.g., adversity, stressors, or demands) that have the potential to disrupt a person’s functioning. As a multi-dimensional process that mediates the effects of stressors on the achievement of positive outcomes, resilience is associated with individual capacities (e.g., self-regulation, cognitive skills, and personality temperament), relationships (e.g., family, friends, peers, and community members), and the availability of community resources and opportunities (e.g., educational, health, recreational and social services) [1–2]. The recent view of resilience as a series of interactions (or transactions) between an individual’s environment and an individual’s assets is a clear departure from previous definitions of resilience, which referred to inherent and static traits within the individual [3–4]. Many scholars have investigated and discussed the interactive, dynamic nature of resilience among young people–including how it is impacted by personal, relational, and contextual factors–a key population that is often seen as particularly vulnerable to engaging in health risk behaviors [5]. As a promoter of positive short- and long-term development, psychological functioning, and physical well-being [6–7], resilience has been connoted as a construct that may protect adolescents from participating in maladaptive, health risk behaviors [8].

Alongside the identification of factors that characterize resilience, researchers have developed instruments to measure the construct. However, previous research has tended to focus on outcomes that (a) emphasize mainstream populations from Westernized, individualistic cultural contexts upon which healthy functioning is defined, and (b) lack sensitivity to community and cultural factors that contextualise how resilience is defined by different populations and manifested in everyday settings [9]. As a result, there has been little cross-cultural validation of resilience within non-western cultures and settings.

Although several instruments have been validated for use with children and youth, the Child and Youth Resilience Measure (CYRM-28 [10]) represents one of the few that has been developed using items generated from a multi-cultural framework and investigator panel and validated using a multi-country sample. Based on validation efforts, a multidimensional, 28-item measure emerged that accounted for the resilience factors–*individual*, *relational*, and *contextual*–that are common across a diverse range of cultural groups [4].

In developing the instrument, however, Ungar and Liebenberg [10] noted several methodological limitations, such as the sole reliance on exploratory factor analysis. The authors advocate the employment of confirmatory factor analysis to continue the construct validation process of the instrument. There were also five items that were retained on the CYRM-28, despite statistical evidence warranting their removal (i.e., failure to load significantly on the first factor in one or more sub-sample). With findings of unique factors, such as the concept of *Batho* (i.e., community interdependence, connectedness, and respect for community values), underlying the resilience process in South African youth [11], it is necessary to determine the relevance of the CYRM-28 structure and item-factor loadings in the South African adolescent context. In fact, recent single-country studies have revealed selected items may be inapplicable in certain contexts [12]. Even though Ungar and Liebenberg’s [10] initial validation included a small group of adolescents from South Africa (*N* = 60), it is unknown whether the sample is representative of, and the results generalizable to, broader South African youth.

The data for this paper was drawn from a longitudinal study (i.e., Youth HIV/AIDS Prevention Project [Y-HAPP]) on factors associated with sexual risk among school going youth in the province of KwaZulu-Natal (KZN), South Africa, which has the highest HIV prevalence rate in South Africa [13]. Often a target of programmatic HIV-focused interventions, resilience is particularly relevant in this context for preventing sexually risky behaviors among adolescents given the high rate of heterosexual transmission of HIV [14]. Although prior research has identified various factors (e.g., school belonging, parental monitoring) act as protectors that reduce the likelihood of sexually risky behaviors among adolescents in this region [15], studies have typically examined protective factors in isolation and have relied on measures that have been validated among Westernized populations [16].

Building on Ungar and Liebenberg’s [10] initial work, this study sought to examine the psychometric properties of the CRYM-28 using a larger sample of South African adolescents. Specifically, we sought to confirm whether the original three-factor, 28-item measure represents the most suitable factor structure, and, using the most parsimonious version of the CYRM in this study, assessed the convergent validity, discriminative ability, and reliability of the instrument.

## Methods

### Participants

#### Time 1

Initial data collection was conducted at seven rural (*N* = 768) and five urban (*N* = 1086) secondary schools within the Bergville Education and Central Durban Education districts, respectively, and each were partner schools on the Y-HAPP project. The Bergville district is characterised by rural, lower income, Black African families (poverty quintiles 1 to 3), whereas the Central Durban district has greater racial diversity and higher socioeconomic status (poverty quintiles 4 and 5). A combination of coeducational and single-sex education schools were included, with the number of purposively sampled participants from each school ranging from 32 to 320. Across the schools, 1854 (*M*_age_ = 14.88 yrs, *SD* = 1.68) adolescent girls (*N* = 900) and boys (*N* = 954) were taken from Grades 8 (*N* = 865) and 10 (*N* = 989). The sample was primarily comprised of Black adolescents (*N* = 1543), followed by Indian (*N* = 139), White, (*N* = 92), Coloured (*N* = 56), and ‘Other’ (*N* = 14) race group participants.

#### Time 2

After a 12-month interval, a total of 648 male (*N* = 324) and female (*N* = 324) participants (*M*_age_ = 15.93 yrs, *SD* = 1.50) from the same seven rural (*N* = 219) and five urban (*N* = 429) schools completed the questionnaire for a second time. The participants were in Grades 9 (*N* = 278) and 11 (*N* = 370), one grade level higher than at time 1. The majority of the time 2 participants were Black (*N* = 520), with Indian (*N* = 58), White, (*N* = 43), Coloured (*N* = 17), and ‘Other’ (*N* = 10) race groups comprising the remainder of the sample.

### Materials

#### Resilience

The Child and Youth Resilience Measure (CYRM-28 [10]) is an indirect measure of resilience; it measures the availability of resources that increase the likelihood of demonstrating resilience when adversity or risk (whatever type or degree) is experienced.

The multidimensional instrument contains 28 Likert-type items (1 = *not at all*, 5 = *a lot*) across three dimensions of resilience: *individual* factors (11 items–Do you strive to finish what you start?), *relational* factors (seven items–Do you think your family will always stand by you during difficult times?), and *contextual* factors (10 items–Do you participate in organized religious activities?).

The three-dimensional structure has been corroborated on several occasions [4, 17]. Researchers have found construct and concurrent validity support for the instrument [12, 18]. Adequate internal consistency has been reported across a number of studies [4, 19] and test-retest reliability estimates have revealed total and subscale score stability over short (i.e., two to five weeks) and long intervals (i.e., three months [4, 12]).

#### School connectedness

Perceptions of school connectedness was measured using the Psychological Sense of School Membership scale (PSSM [20]). The 18-item instrument assesses adolescent school issues related to inclusion, acceptance, respect, encouragement, and sense of belonging (e.g., I feel like a part of my school). The PSSM responses are in the form of a five-point Likert scale ranging from 1 (*not at all true*) to 5 (*completely true*).

Positive relationships with measures of success in school (e.g., grade point average) and hopeful life outcome expectations [21–22], and associations with lower anxiety and depression levels [23] support the concurrent and predictive validity of the instrument. Internal consistency estimates have been acceptable to good (α = 0.76 to .84) across a number of studies [24–26]. In this study, internal consistency was α = 0.72.

#### Parental monitoring

Six items were adapted from the Alabama Parenting Questionnaire-Child Form (APQ [27]) to measure adolescents’ parental monitoring perceptions. The items (e.g., I tell my parents/caregiver who I am going to be with before I go out) are rated on a five-point Likert-type scale anchored at 1 (*never*) and 5 (*very often*). Research has supported the convergent validity of the APQ through associations with antisocial behavioral tendencies and conduct problems [28–29] and the APQ have been found to discriminate children who have conduct problems from those that do not [30]. Subscale internal consistency estimates (alpha) have range from .54 to .83 across a number of studies [28, 31]. Cronbach’s alpha for the six-item scale in this study was .76.

#### Sexual risk

Seven items were used to ascertain the participants’ past and present sexual experiences and combined for a composite sexual risk index. The items measured sexual activity safety (i.e., The last time you had sexual intercourse, did you or your partner use a condom?), number of sexual partners (i.e., In total, how many sexual partners have you had?), age of sexual debut (i.e., How old were you the first time you had sex?), pregnancy incidence (i.e., Have you ever been pregnant or made someone pregnant?), relationships with older partners (i.e., Have you ever had a girlfriend or boyfriend who was more than 5 years older than you?), sexual regret (i.e., Have you ever done something sexual with someone else that you wish you had not done?), and sexual exchanges for gifts (i.e., Have you ever given or received a gift in exchange for sex?). Internal consistency (alpha) for the composite index was .97.

### Procedure

Representatives from a number of schools were first approached in order to explain the purpose of the study and the pupils’ proposed involvement in it. Subsequent to schools’ permission to access students and ethical clearance to conduct the study, written informed consent was obtained from child’s parents/legal guardians. Informed consent was also discussed with the adolescents and written consent obtained prior to them completing the questionnaire. In line with Parry’s [32] recommendations, a specific process of translation, back-translation, and adjustment to assure linguistic equivalence was followed. Two members of the research team, one of whom has a Master’s degree in social science and the other a research officer, translated the questionnaires into IsiZulu. Both were native to the research area and fluent in IsiZulu. Each item was discussed in detail to determine an appropriate translation. A third member of the team who was also fluent in IsiZulu back-translated the items. The adolescents from the Bergville Education district completed questionnaires that were in IsiZulu. The students from the Central Durban Education district requested to complete the English version of the questionnaire. All questionnaires were self-completed under standardised administration conditions. Researchers were available to provide guidance and support to students, when required. Ethical approval for this recruitment and data collection process was granted from the University of KwaZulu-Natal’s Human and Social Science Research Ethics Committee and the Provincial Department of Basic Education, KwaZulu-Natal. On average, data collection lasted two days at each of the schools, and the questionnaire required approximately 30 minutes to complete.

### Data analyses

Measurement model analysis was conducted using AMOS 23, whereas the remaining analyses were computed using SPSS 23. The number of missing values for each CYRM-28 item was less than 2% (i.e., .3% to 1.5%); missing values were subsequently replaced using expectation maximisation [33]. Although maximum likelihood estimation with large sample sizes (> 500 [34]) is quite robust to normality assumption violations [35], hypothesis testing assumptions were examined before conducting confirmatory factor analysis using the approach. General recommendations are that, in addition to chi-square (χ^2^), a number of incremental and absolute fit indices should be used to assess model fit [36]. These indices are particularly important to consider, as χ^2^ *p*-values will often achieve significance when models are estimated using large sample sizes [37]. In this study, incremental fit indices [Tucker-Lewis index (TLI) and comparative fit index (CFI)], absolute fit indices [standardized root mean square residual (SRMR) and root mean square error of approximation (RMSEA)], and a comparative fit index [Akaike Information Criterion (AIC)], were examined. Incremental fit indices compare a baseline model (i.e., all variables are uncorrelated) to a specified model, absolute fit indices provide an indication of how adequately the specified model fits the data, and comparative fit indices enable comparisons to be made between tested models [38].

Although normative criteria for incremental and absolute fit indices have been proposed, researchers caution against stringent and absolutist application of fit index guidelines [39]. The fit indices should also be considered in conjunction with one another and model fit decisions based on any single index should be avoided [40]. Among the most commonly used fit index recommendations are > .90 for CFI and TLI, < .08 for SRMR, and < .06 for RMSEA [39, 41–42]. AIC values are compared across models, with lower values indicating a superior fitting model. There are several important caveats to applying fit index rules of thumb. Rigdon [43] suggests RMSEA is better suited for confirmatory analyses in which the purpose is to determine the adequacy of an established model, and the CFI is most appropriate for exploratory purposes in which the goal is to determine the ‘best’ fitting model. When RMSEA values approach .05 and RMSEA null values are less than .158, CFI and TLI estimates are uninformative and unlikely to achieve values above .90 [44–45]. There is also evidence that the CFI index tends to reject correctly specified models when factor loadings are low-to-medium, which is often the case with psychological research [46].

The relationships between the latent factors were also explored, and Cronbach’s was alpha computed for each of the scales at time 1. Alpha criteria of excellent (> .90) good (> .80), acceptable (> .70), questionable (> .60), poor (> .50), and unacceptable (< .50) were used to assess internal consistency [47]. Subscale test-retest reliability was performed using intraclass correlation coefficients (ICC), which are more appropriate than Pearson’s correlations when group means are comparable and there is individual score variability across data collection points [48]. Mean subscale score differences were tested using paired-samples *t*-tests.

The convergent validity of the CYRM scales was tested using adolescents’ self-reported parental monitoring and school connectedness perceptions, whereas criterion validity was examined using a measure of sexual risk. Sex and age group comparisons were performed to determine the discriminative ability of the scales. With under 15 years of age an important benchmark for identifying adolescents at risk (e.g., early sexual debut [49]), age was dichotomised into younger than 15 years and 15 years and older groups. To preserve familywise alpha, Bonferroni adjustments were applied to follow-up univariate analyses.

## Results

### Confirmatory factor analysis

The factor-level analysis results for the three models are presented in Table 1. The χ^2^ exact fit index was statistically significant for each model (*p* < .001), but the RMSEA and SRMR values indicated a good fit for each model. The null RMSEA value for each model was below the threshold of .158, and, when RMSEA values approach .05, it is questionable whether CFI and TLI could produce values above .90 [45]. Therefore, the interpretability of these fit indices are limited in this study. Also, with CFI estimates tending to indicate model rejection with low-to-medium item-factor loadings [46], the loadings in this study (see Table 2) may account for the substandard CFI index for each model. Collectively, the results suggest a reasonable level of fit for each model, but the best fit was found for Model 2. Comparing the fit indices and the standardized item factor loadings for each model, the three-factor, 24-item structure (hereafter referred to as CYRM-24) offers the most parsimonious measurement model.

**Table 1:** Summary of fit indices for measurement models.

Model	χ^2^	*df*	*p*-value	CFI	TLI	AIC	SRMR	RMSEA [90% CI]	*p* close	RMSEA null model
Model 1: Original three factors (28 items)^a^	2557.60	347	< .001	.832	.816	2675.60	.051	.059 [.057, .061]	< .001	.137
Model 2: Three factors (24 items)^b^	1567.55	249	< .001	.880	.867	1669.55	.042	.053 [.051, .056]	.012	.146
Model 3: Four factors (28 items)^c^	2419.94	344	< .001	.842	.826	2543.94	.046	.057 [.055, .059]	< .001	.137

Note.

^a^ Model 1: Original three-factor model including all 28 items (Liebenberg et al., 2012; Ungar & Liebenberg, 2011).

^b^ Model 2: Three-factor model including 24 items (Daigneault et al., 2013).

^c^ Model 3: Four-factor model including all 28 items (Sanders et al., 2015).

**Table 2:** Standardized CYRM item-factor loadings for all tested models.

CYRM Items	Model 1^a^	Model 2^b^	Model 3^c^
1. Do you have people you look up to?	.41^C^	-	.43^SO^
2. Do you cooperate with people around you?	.52^I^	.50^I/S^	.53^I^
3. Is getting an education important to you?	.49^C^	-	.48^SO^
4. Do you know how to behave in different social situations?	.55^I^	.53^I/S^	.54^SO^
5. Do you feel that your parent(s) watch you closely?	.49^R^	-	.49^F^
6. Do you feel that your parent(s) know a lot about you?	.58^R^	.56^F^	.59^F^
7. Do you eat enough most days?	.56^R^	.57^F^	.57^F^
8. Do you strive to finish what you start?	.48^I^	.47^I/S^	.48^I^
9. Are spiritual beliefs a source of strength for you?	.50^C^	.52^C/S^	.53^SP^
10. Are you proud of your ethnic background?	.55^C^	.56^C/S^	.53^SO^
11. Do people think you are fun to be with?	.58^I^	.58^I/S^	.59^I^
12. Do you talk to your family about how you feel?	.50^R^	.51^F^	.51^F^
13. Are you able to solve problems without using illegal drugs and/or alcohol?	.41^I^	.38^F^	.42^I^
14. Do you feel supported by your friends?	.59^I^	.60^I/S^	.60^I^
15. Do you know where to go in your community to get help?	.49^I^	.51^I/S^	.49^SO^
16. Do you feel you belong at your school?	.46^C^	.46^I/S^	.45^SO^
17. Do you think your family will always stand by you during difficult times?	.63^R^	.63^F^	.63^F^
18. Do you think your friends will always stand by you during difficult times?	.58^I^	.60^I/S^	.59^I^
19. Are you treated fairly in your community?	.51^C^	.55^I/S^	.52^SO^
20. Do you have opportunities to show others that you are becoming an adult?	.58^I^	.59^I/S^	.56^SO^
21. Are you aware of your own strengths?	.61^I^	.60^I/S^	.61^I^
22. Do you participate in organized religious activities?	44^C^	.49^C/S^	.53^SP^
23. Do you think it is important to serve your community?	.51^C^	.54^C/S^	.58^SP^
24. Do you feel safe when you are with your family?	.61^R^	.61^F^	.61^F^
25. Do you have opportunities to develop job skills that will be useful later in life?	.52^I^	.53^C/S^	.51^SO^
26. Do you enjoy your family’s traditions?	.52^R^	.51^F^	.51^F^
27. Do you enjoy your community’s traditions?	.47^C^	.50^C/S^	.53^SP^
28. Are you proud to be South African?	.33^C^	-	.30^SO^

Note.

^a^ Model 1: Original three-factor model (I = Individual, R = Relational, C = Contextual) including all 28 items (Liebenberg et al., 2012; Ungar & Liebenberg, 2011).

^b^ Model 2: Three-factor model (I/S = Individual/Social, F = Familial, C/S = Community/Spiritual) including 24 items (Daigneault et al., 2013).

^c^ Model 3: Four-factor model (SO = Social/Cultural, F = Family, I = Individual, SP = Spiritual/Community) including all 28 items (Sanders et al., 2015).

### Reliability

Cronbach’s alpha coefficients revealed internal consistency was good for the CYRM-24 *individual/social* scale (α = .82) and acceptable for the *familial* (α = .71) and *community/spiritual* (α = .70) scales. The ICC computations were above .60 for each scale (see Table 3), supporting the temporal stability of the scores from time 1 to time 2 [50]. The paired-samples *t*-tests revealed a significant time 1 to time 2 difference on the *individual/social* scale (*p* = .015) but not on the *familial* (*p* = .443) and *community/spiritual* (*p* = .196) scales. However, applying Cohen’s [51] effect size standards, each of the effect sizes was small (*d* = .03 to .10). On the whole, the results support the reliability of the three scales.

**Table 3:** Baseline to 12-month intraclass correlation coefficients and paired-samples t-tests.

Variable	Time 1	Time 2	ICC^d^	ICC 95% CI		*t*^e^	*p*-value	*d*
				Lower	Upper			
Individual/Social			.62	.55	.67	-2.44	.015	.10
* M*	42.08	42.89						
* SD*	8.40	7.68						
Familial			.63	.56	.68	-.77	.443	.03
* M*	29.70	29.85						
* SD*	5.07	4.62						
Community/Spiritual			.65	.59	.70	-1.29	.196	.05
* M*	23.10	23.35						
* SD*	4.90	4.79						

Note.

^d^ ICCs represent average measures based on two-way random effects models with absolute agreement specified.

^e^ *df* = 647.

### Construct validity

Supporting the convergent validity of the CYRM-24 scales, the *individual/social*, *familial*, and *community/spiritual* scales correlated positively with school connectedness and parental monitoring, all of which were medium in effect size (see Table 4). The descriptive statistics for the MANOVA analyses are presented in Table 5. The distribution for the sexual risk index was bimodal, resulting in the dichotomization of the variable into low and high risk. The MANOVA for sexual risk was significant, *F* (3, 1738) = 8.98, *p* < .001, Wilk's Λ = .985, $\eta_{p}^{2}$ = .015, with differences found on the *individual/social* [*F* (1, 1740) = 6.30, *p* = .036, $\eta_{p}^{2}$ = .004], *familial* [*F* (1, 1740) = 25.12, *p* < .001, $\eta_{p}^{2}$ = .014], and *community/spiritual* [*F* (1, 1740) = 9.21, *p* = .006, $\eta_{p}^{2}$ = .005] between those categorised into low and high sexual risk.

**Table 4:** Pearson correlations between the CYRM, school connectedness, and parental monitoring scales.

**Variable**	**(1)**	**(2)**	**(3)**	**(4)**	**(5)**
(1) Individual/social	-	.69*	.64*	.41*	.44*
(2) Familial	-	-	.62*	.29*	.45*
(3) Community/spiritual	-	-	-	.37*	.37*
(4) School connectedness	-	-	-	-	.20*
(5) Parental monitoring	-	-	-	-	-
*N*	1854	1854	1854	1500	1451
*M*	41.39	29.36	22.92	55.82	25.47
*SD*	8.67	5.23	5.03	9.82	6.31

Note.

* *p* < .001.

**Table 5:** Descriptive statistics for CYRM-24 subscales by sexual risk, sex, and age groupings.

	Individual/social		Familial		Community/spiritual	
	*M*	*SD*	*M*	*SD*	*M*	*SD*
Sexual risk						
Low risk (*N* = 1393)	41.75*	8.77	29.74*	5.12	23.14*	5.08
High risk (*N* = 349)	40.46	8.04	28.19	5.33	22.23	4.83
Sex						
Male (*N* = 954)	41.61	8.84	29.20	5.35	22.63	5.22
Female (*N* = 900)	41.16	8.47	29.52	5.09	23.22*	4.80
Age						
Younger than 15 years (*N* = 787)	41.93	8.85	29.74*	5.23	23.23	5.15
15 years and older (*N* = 1066)	41.00	8.51	29.08	5.21	22.68	4.92

Note.

* *p* < .05 (greater resilience subscale scores).

The MANOVA for sex indicated significant male and female differences, *F* (3, 1850) = 7.45, *p* < .001, Wilk's Λ = .988, $\eta_{p}^{2}$ = .012, although this pertained strictly the *community/spiritual* scale [*F* (1, 1852) = 6.57, *p* = .030, $\eta_{p}^{2}$ = .004] and not the *individual/social* [*F* (1, 1852) = 1.25, *p* = .792, $\eta_{p}^{2}$ = .001] or *familial* [*F* (1, 852) = 6.30, *p* = .036, $\eta_{p}^{2}$ = .004] scales. Although the MANOVA for age group was significant, *F* (3, 1849) = 2.69, *p* = .045, Wilk's Λ = .996, $\eta_{p}^{2}$ = .004, univariate analyses indicated differences on the *familial* [*F* (1, 1851) = 7.27, *p* = .021, $\eta_{p}^{2}$ = .004] scale, but not the *individual/social* [*F* (1, 1851) = 5.18, *p* = .069, $\eta_{p}^{2}$ = .003] or *community/spiritual* [*F* (1, 1851) = 5.45, *p* = .060, $\eta_{p}^{2}$ = .003] scales. Collectively, the selected sexual risk, age, and sex group differences were generally small to medium in effect size [52]. When significant, subscale scores were higher for the low sexual risk, female, and youth younger than 15 years of age (see Table 5).

## Discussion

The purpose of this study was to examine the psychometric properties of the CYRM-28 in a sample of South African adolescents in a high HIV prevalence setting. This study, therefore, builds on both the initial development and subsequent validation of the instrument in more developed countries [4, 10, 53]. The results showed that the original CYRM-28 revealed a reasonable level of fit, however, a superior level of fit was found for three-factor–*individual/social*, *familial*, *community/spiritual*– 24-item model (items 1, 3, 5, and 28 removed) reported by Daigneault et al. [12]. This may be because selected items, such as item 5 (i.e., Do you feel that your parent(s) watch you closely?), were among the exceptional items retained on the CYRM-28. Also, item 28 (i.e., Are you proud to be South African?) assesses national pride, which is much broader in scope than the other items included on Liebenberg et al.’s [4] *contextual* subscale. The finding of an improved factor structure with these items removed is not isolated to this study, as previous researchers have also revealed problematic factor loadings in previous validation studies using the original English version of the scale, particularly for items 1, 5 and 28 [4, 54]. Sexual risk prevention research relies substantially on determining the availability of protective factors in this region, which can be leveraged to target those at risk for sexually transmitted diseases [55]. Given high HIV prevalence rates among adolescents and young women in the region [56], the findings in this study may coalesce the diversity in resilience measurement in South Africa [9] and facilitate the targeting of resilience resources that are relevant and available to these key populations.

The results in this study further showed that the CYRM-24 and its subscales are internally consistent. The Cronbach’s alpha values for each of the CYRM-24 scales were similar to those reported by previous validation studies [4, 12], and the test-retest reliability estimates support the consistency of the scales over an interval of 12 months. The findings also indicated positive correlations between all the CYRM-24 subscales, parental monitoring, and sense of school connectedness. This indicates that adolescents with greater features of resilience also reported greater perceived parental monitoring and a stronger sense of school connectedness. These findings corroborate the results of other studies that have linked resilience to good parenting practices and school belongingness [57–59].

In support of the discriminative power of the revised 24-item version, a difference between age group was observed on the *familial* subscale, with younger adolescents having greater familial resilience than older adolescents. This finding may reflect the developmental phase of adolescents, as younger adolescents are more likely to be comfortable and safer when they are with their families. Older adolescents, on the other hand (particularly those over 15 years), are more likely to associate with their peers [60]. Females were found to score higher on the *community/spirituality* subscale. With the items on this subscale measuring aspects related to emotional and social strength, attributes that favour adolescent girls [61], this may account for the difference found between males and females. All three subscales of the CYRM-24 were associated with low sexual risk behaviors, with participants reporting higher levels of resilience scoring lower on the sexual risk index. This is consistent with suggestions that resilience among young adolescents may serve as a protective factor against health risk behaviors [8]. The finding provides support for the protective model of resilience, which posits that protective factors assist in neutralizing the effect of risk, thereby reducing the likelihood of maladaptive consequences [58].

### Strengths and limitations

This is one of the first studies to provide preliminary evidence on the suitability of the CYRM-28 in the South African context, a setting in which resilience has historically been measured using instruments that have been validated in Western populations [9]. Based on the psychometric support received for the CYRM-24 in this study, the revised version represents a culturally and contextually-specific measure of resilience among South African adolescents. This is likely to benefit the application and measurement of resilience in programmatic interventions that target sexual risk within high HIV prevalence regions such as KwaZulu-Natal. Despite these strengths, key limitations should be noted. Although the study includes a cross-ethnic analysis, the sample includes South African adolescents from schools located within two educations districts in a single province. As such, replication research appears necessary with samples of youth, both nationally internationally, in order to ascertain and maintain the instrument’s distinction as a cross-culturally relevant measure of resilience.

Given the different theoretical perspectives on resilience evident in the literature [62], this study adopted the view that resilience in all people is an interactive process involving varying degrees of risk and the availability of resources that can be accessed to serve as protective mechanisms for the individual. This view informed the decision not to exclusively focus on so-called ‘high-risk’ individuals (i.e., those reporting high-risk sexual behaviors) in the analysis. Further, the heterogeneous nature of our sample (i.e., adolescents with different levels of sexual risk behavior) was beneficial for assessing the broad applicability of the instrument for South African adolescents. Another strength of the study was the inclusion of a large sample. However, the participants were conveniently sampled, which has generalisability implications. It should also be noted that, given the content of some questionnaire items (e.g., sexual risk), there may have been a tendency for the participants to respond in a socially desirable manner. Therefore, caution should be applied when interpreting the results that are based on indexes containing such items.

## Conclusions

This study examined the psychometric properties of the CYRM-28 in a sample of South African adolescents. The findings supported a superior, revised, three-factor structure comprising 24 items (CYRM-24), with each factor representing the *individual/social*, *familial*, and *community/spiritual* dimensions. Convergent validity support was evidenced in the positive associations between the CYRM-24 scales, school connectedness, and parental monitoring. An appropriate level of reliability was found for each scale, and selected scales were able to discriminate between sex, age, and sexual risk groupings. Collectively, the results suggest the CYRM-24 is a valid and reliable measure of adolescent and youth resilience, though additional research may be required to validate the instrument for use with multiple South African sub-groups and to determine whether further refinements to the CYRM-24 are required.

## Supporting information

S1 File Primary dataset. (SAV)

S2 File Test-retest reliability dataset. (SAV)
